# Research on Evaluation Index System of Chinese City Safety Resilience Based on Delphi Method and Cloud Model

**DOI:** 10.3390/ijerph16203802

**Published:** 2019-10-09

**Authors:** Jingjing Pei, Wen Liu, Lu Han

**Affiliations:** School of Engineering & Technology, China University of Geosciences-Beijing, Beijing 100083, China; peijj@cugb.edu.cn (J.P.); 13051827622@163.com (L.H.)

**Keywords:** city safety resilience, indicators, evaluation system, Delphi method, cloud model

## Abstract

To scientifically and quantitatively evaluate the current city safety resilience and improve the city safety resilience level, this project puts forward the concept and degree of city safety resilience based on the systematic analysis of the city safety resilience curve and establishes the framework of a city safety resilience evaluation index system, including predisaster prevention, disaster-bearing carrier, emergencies and emergency management. The Delphi method and cloud model are used to construct the city safety resilience evaluation model, and the weight and application of the model are analyzed and discussed. The research results show that the theory and method of Chinese city safety resilience evaluation based on the Delphi method and cloud model have important guiding significance for improving the city safety resilience level.

## 1. Introduction

It is estimated that 60% of the world’s population is destined to live in medium–large cities by 2050 [1]. However, safety has become an increasingly important issue in modern city planning. Economic and social activities in Chinese cities are intensive and diverse, and include hazardous substances or energy. Meanwhile, production facilities and processes are becoming increasingly complex, comprising the unique risk characteristics of city production. Reported natural disasters, such as storms, tropical cyclones and floods, have led to a gradual increase in global economic losses due to increased population and risk to capital [2]. It is undeniable that catastrophic consequences such as the loss of life and property and the failure of city functions are common. For example, the “8.12” fire and explosion accident of dangerous chemicals in Tianjin Port resulted in 165 deaths and 6.6 billion RMB in property losses. Typhoon Skypigeon caused 24 deaths and $4.8 billion in property damage in several cities in August 2017.

In these cases, the introduction of resilience engineering undoubtedly provides a new way of thinking and a direction for dealing with the crisis and risk of city production safety and natural disasters. It covers the major fields of social and physical factors such as the economy, the environment and technology; it covers the whole pre-prevention, in-process control and postemergency management process; and it emphasizes the city’s ability to resist, absorb, adapt and recover from public safety incidents. As Branscomb [3] describes, city areas are the most important social infrastructure and have corresponding flexibility. They are often more vulnerable to three types of disasters: technical disasters (caused by human error and infrastructure failure (such as power failure), natural disasters (such as hurricanes, floods, earthquakes, tsunamis), and terrorism. Considering the research direction of the Ministry of Emergency Management of China, this paper focuses on the evaluation system of city safety resilience, focusing on the production safety and city natural disasters extended by technical disasters. Therefore, the purpose of this paper is to increase the ability of urban resistance accidents through effective evaluation of urban safety level, so as to provide a theoretical basis for strategic thinking and countermeasures in resilience city construction.

Scholars in various countries have begun to refine the evaluation of city resilience into specific indicators, and to use quantitative indicators to evaluate the ability and degree of city resilience scientifically, so that the design of city resilience evaluation systems tends to be comprehensive and scientific. For example, Stanton and Miner [4] believe that community resilience mainly includes four aspects: infrastructure elasticity, institutional elasticity, economic elasticity and social elasticity. Comfort [5] mentioned that information technology should be combined with organizational learning when discussing how to improve community disaster reduction capacity. Twigg [6] emphasizes that when defining flexible community, the concept of community should include multiple dimensions such as common interests, values, behavior structure, etc. Cimellaro [7] set up a framework for evaluating cities from seven dimensions. The indicator system proposed by the Regional Institute of Buffalo University and Rockefeller Foundation is widely recognized, internationally. It describes city resilience in four dimensions: health, economy and society, urban system and services, leadership and strategy [8]. Domestic research on resilience started relatively late, and the research hotspots at the city level have been focused on the resilience of city water systems represented by sponge cities, and have mainly been focused on comprehensive research. Liu Jiangyan [9] and other research into foreign resilient cities was used for reference, and domestic research was constructed on the basis of four aspects: ecological resilience, economic resilience, engineering resilience and social resilience. Guo Xiaodong [10] and others took the ratio of community resilience and vulnerability of community disasters as the criterion of resilience evaluation, and constructed an evaluation model of community resilience. In 2018, Fan Weicheng [11] and others put forward a triangular model of urban safety resilience, which consists of three sides: public safety events, urban disaster-bearing systems, and safety resilience management. From the above, it can be seen that the concept of urban safety resilience has not yet formed a unified understanding in the academic circles, and the theoretical and applied research is still relatively deficient.

On this basis, the research structure of this paper is as follows: Section 2 explores the framework of city safety resilience evaluation index system according to the definition and characterization model. Section 3 presents the methods used in this study—the Delphi method and the cloud model method. Section 4, based on the framework of urban safety toughness evaluation system, uses the Delphi method and the cloud model method to construct the evaluation model of city safety resilience, and obtains the weight of each index. The fifth section analyzes and discusses the weight and application of the city safety resilience evaluation model. Finally, in the sixth part, the research results are discussed and suggestions for future research are put forward.

## 2. Establishment of Evaluation System Framework of City Safety Resilience

### 2.1. City Safety Resilience

The definition of resilience has not been unified. Nevertheless, the research object extends from simplifying abstract ecosystems or traditional engineering systems to complex multistable systems, and has been gradually extended to various fields. Its core is to maintain and restore its normal function using responses, absorption, maintenance and restoration. Cities are the cornerstones of global economic and cultural activities. City safety ensures the normal operation of city functions. The high correlation between city safety resilience engineering means that improved city safety resilience can maintain the normal functional level of a city and recover to the expected functional level in a shorter time after being impacted.

The U.S. Department of Homeland Security defines city resilience as “the ability of social property, social space, or city networks to recover their functions in case of terrorist attacks or other incidents”. The widely accepted concept of “city resilience” is that the city system can withstand significant damage within acceptable degradation parameters, absorb some of the destructive energy, and restore the original city capacity level within an acceptable time. Desouza [12] pointed out in 2013 that safety resilience refers to the ability of city systems to absorb, adapt and respond to changes. In 2016, Meerow [13] pointed out that city safety resilience refers to the ability of a city system and its social-ecological and Social-Technological network to maintain and quickly restore the required functions in the face of disturbances in order to adapt to the rapid transformation ability of current or future changes with respect to both time and space. Based on the two directions of city natural disasters and production safety, this paper defines city safety resilience as the ability of the whole city system to absorb a large amount of energy that is released by disasters, reduce the losses that are caused by disasters, and quickly restore the original functional level when the whole city system is affected by emergencies.

### 2.2. Characterization Model of City Safety Resilience

**Figure 1:**
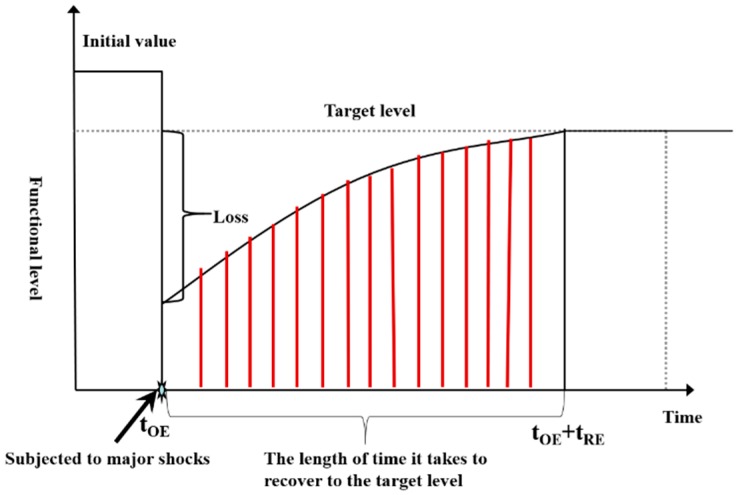
Safety resilience curve.

This paper modifies the city public safety resilience curve of Xu Hui [14] and introduces the city safety resilience curve, as shown in Figure 1.

The vertical axis of the image is the function level of the city when it is operating normally, and the horizontal axis is time. The image reflects the target city’s safety resilience level, which can maintain the normal operations of a city area in a changing environment and can resist risk events to restore city functions.

Starting from the concept of the safety resilience curve, the researcher takes into account factors such as the system state, recovery time and so on, and characterizes city safety resilience using a mathematical definition, as shown in Formula (1):

(1) $$R\left(\vec{r}\right)=\int_{t_{OE}}^{t_{OE}\;+t_{RE}}Q(\vec{r},t)dt$$

where $\vec{r}$ is the space vector defining the position. $R\left(\vec{r}\right)$ is the resilience value of the whole system after the occurrence of risk events. $t_{OE}$ is the moment when a risk event occurs. $t_{RE}$ is the recovery time. $Q(\vec{r},t)$ is the ability to recover after a risk event.

### 2.3. Model Construction

From the point of view of national policies, laws and regulations, this study collects the relevant literature from at home and abroad. According to the characteristics of the optimized resilience curve, the theory of modern city safety space construction [15] and the triangular model of public safety put forward by Fan Weicheng [10], it divides a city safety resilience system into predisaster prevention, disaster-bearing carriers, emergencies and emergency management. $A_{i}$ represents the index of this level, i=1,2,3,4. To make the strategy and countermeasure for improving city safety resilience more specific and detailed, the secondary and tertiary index factors covered by city safety resilience index are selected preliminarily, and the main factors affecting city safety resilience are collected and selected from policies, laws and regulations, as well as the relevant literature, according to Table 1. $B_{j}$ is used to represent the two-tier index, *J* = 1, 2, …, 12, and $C_{k}$ is used to represent the three-tier index, *k* = 1, 2, …, 37.

#### 2.3.1. Predisaster Prevention ($A_{1}$)

**Figure 2:**
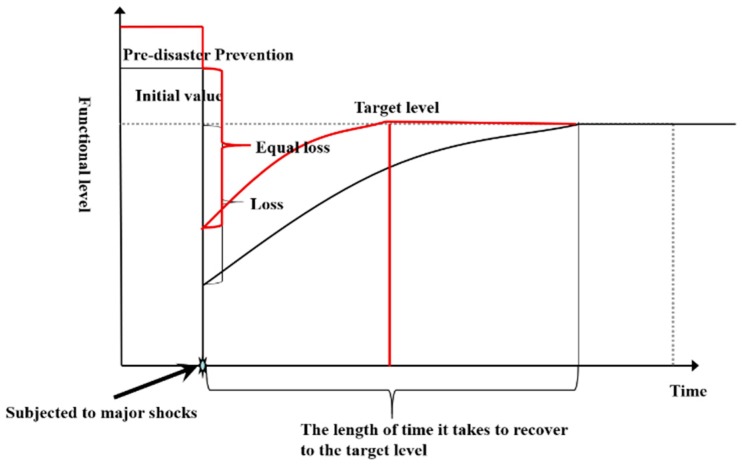
Contrast chart of city safety resilience curve before and after disaster prevention.

The Opinions of the State Council of the Central Committee of the Communist Party of China on Promoting the Reform of the System and Mechanism of Disaster Prevention, Mitigation and Relief [18] clearly state that we should attach great importance to predisaster prevention and improve the predisaster prevention measures. Many organizations and institutions abroad, such as the United Nations, the World Bank, insurance companies and the Red Cross, have also focused their attention on predisaster prevention planning [24]. Predisaster prevention occurs mainly through the continuous monitoring of potential threats and the implementation of disaster prevention planning or risk control by relevant departments, regular and strict disaster prevention training by grass-roots units, the integration of rescue resources, and the utilization of city safety equipment. In the case of the same losses, the cities that have been managed after the predisaster prevention can recover to their original levels quicker at the same recovery rate, as shown in Figure 2.

Predisaster prevention is generally divided into (1) government supervision and prevention ($B_{1}$), which includes the city safety supervision department ($C_{1}$), the active cooperation of different departments ($C_{2}$), the city safety management laws and regulations ($C_{3}$), comprehensive emergency monitoring and risk assessment measures ($C_{4}$) and a risk reduction plan ($C_{5}$); and (2) grassroots city safety prevention ($B_{2}$). This mainly includes the following indicators: emergency prevention regulations ($C_{6}$), the propaganda of emergency prevention ($C_{7}$) and the categories and quantities of safety equipment and facilities ($C_{8}$). Through the active cooperation of various regulatory departments, a series of regulations have been issued to prevent disasters and enhance city safety resilience.

#### 2.3.2. Disaster-Bearing Carrier ($A_{2}$)

**Figure 3:**
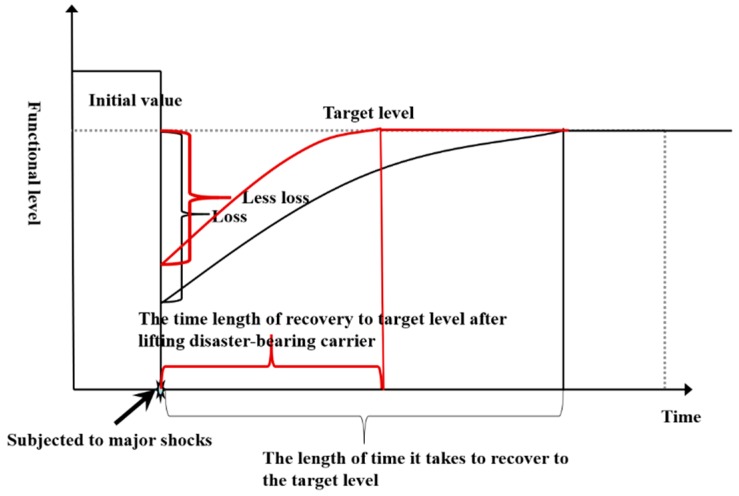
Contrast chart of different disaster-bearing vehicles on the city safety resilience curve.

According to the triangular model of public safety, the disaster-bearing carriers are the objects of natural disasters and production accident disasters. The main disaster-bearing carriers in a city system are the people and the main buildings in the city. The function of a disaster-bearing carrier in the resilience curve is mainly to absorb the effects that result from emergencies [25]. From Figure 3, we can see that if the disaster bearing capacities of city carriers are better, the impacts of emergencies on the original function level of the city will be smaller, and the loss will be smaller. For the same city, under the same recovery rate, the carrying capacity of the city carrier is higher and can return to the target level faster.

The following disaster bearing capacities of the carrier should be considered: (1) Infrastructure ($B_{3}$), including the completeness of the flood control and drainage system ($C_{9}$), the road traffic volume and accessibility ($C_{10}$) and the lifeline resilience ($C_{11}$); (2) city safety facilities ($B_{4}$), including the number of hospitals ($C_{12}$), fire protection coverage ($C_{13}$) and the number of city shelters ($C_{14}$); and (3) housing buildings ($B_{5}$), including the number of anti-seismic buildings ($C_{15}$) and the number of old residential areas ($C_{16}$). Furthermore, we need to consider (1) the population cluster statistics ($B_{6}$), including the population cluster locations ($C_{17}$) and the population density ($C_{18}$) to quantify the population cluster and (2) crowd safety protection measures ($B_{7}$), including the crowd evacuation guidelines ($C_{19}$) and the crowd safety facilities ($C_{20}$).

#### 2.3.3. Emergencies ($A_{3}$)

**Figure 4:**
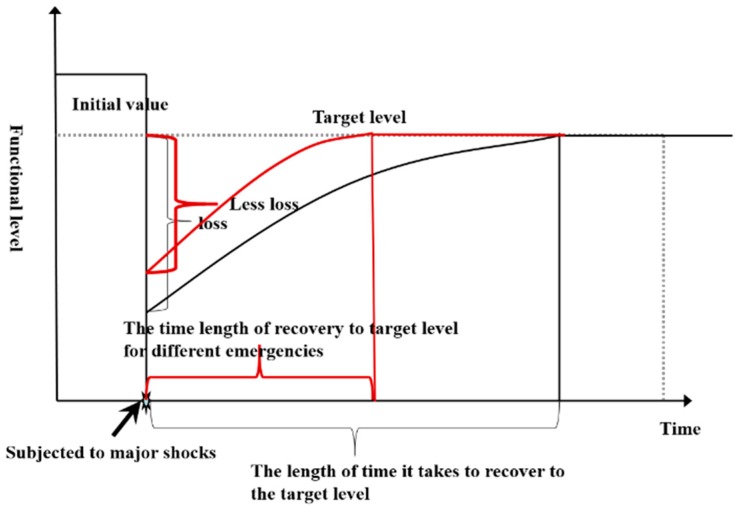
Contrast chart of how different emergencies affect the city safety resilience curve.

The Law of the China on Emergency Response points out that emergencies are natural disasters, accidents, public health incidents and social safety incidents that suddenly occur, may cause serious social hazards and need to be dealt with using emergency measures [26]. However, the object of this study is only natural disasters and accidents. Different cities face different emergencies. For example, Guangzhou is often affected by typhoons, and Shanxi is greatly impacted by coal mining accidents. Different emergencies cause different losses for cities, and the city safety resiliences are also different. This paper compares the impacts of different emergencies on the city safety resilience curve. For cities with the same recovery speed, the smaller the impact of emergencies on the functional level of the city is, the faster that the city can be restored to the target level, as shown in Figure 4.

When defining the losses that are caused by emergencies, the following indicators are used: (1) natural disasters ($B_{8}$), including the frequency of natural disasters in city areas ($C_{21}$), property losses ($C_{22}$) and casualties ($C_{23}$); and (2) production accidents ($B_{9}$), including the frequency of production accidents ($C_{24}$) caused by natural disasters in cities every year, the mortality rate per 100,000 people as a result of production accidents ($C_{25}$), the accident GDP per 100 million yuan based on the mortality rate ($C_{26}$) and production accident economic losses ($C_{27}$).

#### 2.3.4. Emergency Management ($A_{4}$)

**Figure 5:**
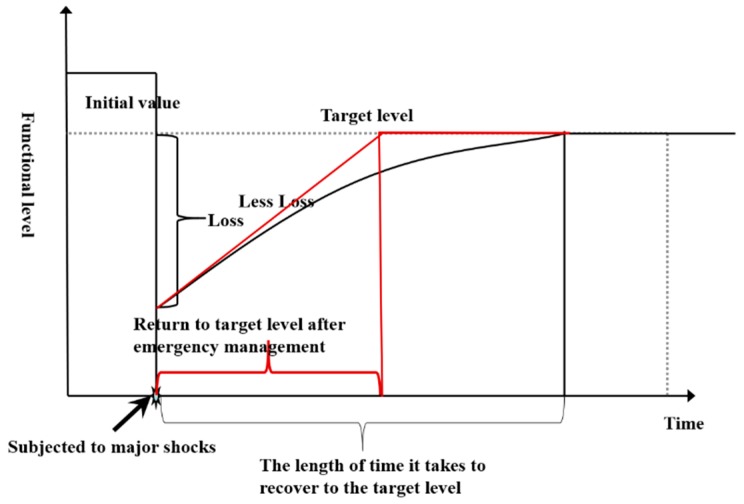
Impact comparison of the city safety resilience curve after emergency management.

Emergency management refers to a series of policies or measures that are adopted by the government and other public organizations to prevent, manage and reduce the impacts of disasters to protect public life, health and property, maintain social stability and improve city safety resilience. Emergency management is an important manifestation of city safety resilience and a response mechanism for emergencies. After being affected by emergencies, the functions of the whole system are damaged and some of them are lost. If emergency management is improved, it can more quickly restore the functional level of the target after city safety is threatened, as shown in Figure 5. However, due to overlapping concepts, the emergency management indicators in this study focus on the processes after emergencies.

For emergency management, this includes the following indicators: (1) the emergency plan ($B_{10}$), the city emergency plan ($C_{28}$), and the city emergency related laws and regulations ($C_{29}$); (2) the emergency management mechanism ($B_{11}$), including the decision-making and disposal mechanism ($C_{30}$), the information reporting mechanism ($C_{31}$), the emergency linkage mechanism ($C_{32}$), the recovery and reconstruction mechanism ($C_{33}$); and (3) emergency support ($B_{12}$), which includes the number of city professional rescue workers ($C_{34}$), the frequency of emergency drills ($C_{35}$) and the city rescue material reserves ($C_{36}$).

## 3. Methodology

### 3.1. Delphi Method

The Delphi method was developed by the Rand Company in the early 1950s and introduced in 1975. It is widely used to obtain opinions and suggestions from expert groups with relevant professional backgrounds [27]. This research has four main characteristics: anonymity, iteration, controlled feedback and expert feedback. Round iteration is the key point of the Delphi method, which is used to identify the differences among experts’ opinions in the whole process, and finally reach consensus. When using the Delphi method, the following four points are emphasized:

(1) The information between experts is not transparent, so as to avoid authority in the industry influencing the judgment of other experts;
(2) Experts are allowed to change their opinions after each round is completed;
(3) The results of each round should be anonymously fed back to experts to ensure in-depth discussion among them;
(4) The statistical analysis of the final feedback should take into account the feedback from each expert, and each statistical result should be mentioned, so as to avoid the shortcoming that the expert meeting method only reflects the views of the majority.

Data were obtained by three-step Delphi method:

Firstly, in order to determine the validity of the framework of city safety resilience evaluation system and the weight of each index, the questionnaire was compiled according to the index factors of the framework, and the respondents were asked to fill in the weight of each index. The weights of the questionnaire ranged from 0 to 1, which was divided into five files. Indicators were not important: [0–0.2], indicators were slightly important: [0.2–0.4], indicators were more important: [0.4–0.6], indicators were very important: [0.6–0.8], indicators were absolutely important: [0.8–1]. When scoring the weights of secondary and tertiary indicators, only the importance of the upper level was considered. No comparison was made between the indicators at each level.

Secondly, a questionnaire on the preliminary index system of city safety was distributed to 15 industry experts, requiring them to have relevant professional background, rich industry knowledge and practical experience. Experts were included of theoretical workers with professional titles, emergency management staff and senior safety management personnel of enterprises.

Thirdly, a questionnaire survey was conducted to allow experts to score according to their own experience and preferences.

### 3.2. Cloud Model Method

The cloud model [28,29] is a cognitive model based on a normal distribution function and a membership function. It realizes the bidirectional transformation between qualitative concepts and quantitative data based on probability statistics and fuzzy set theory. It can effectively represent the concepts of fuzziness, randomness and uncertainty, and has been applied in many fields such as weight calculations, time series mining, decision-making and evaluations.

Suppose that U is a universe that is expressed by exact values, *C* is a qualitative concept in the universe U, and the random value *x* satisfies x∈U. *x* to *C* in the degree of certainty μ should satisfy μ(x)∈ [0, 1]. Then, *x* and its distribution in U are called cloud droplets and clouds, respectively.

(2) μ:U→[0, 1] ∀x∈U x→μ(x)

**Figure 6:**
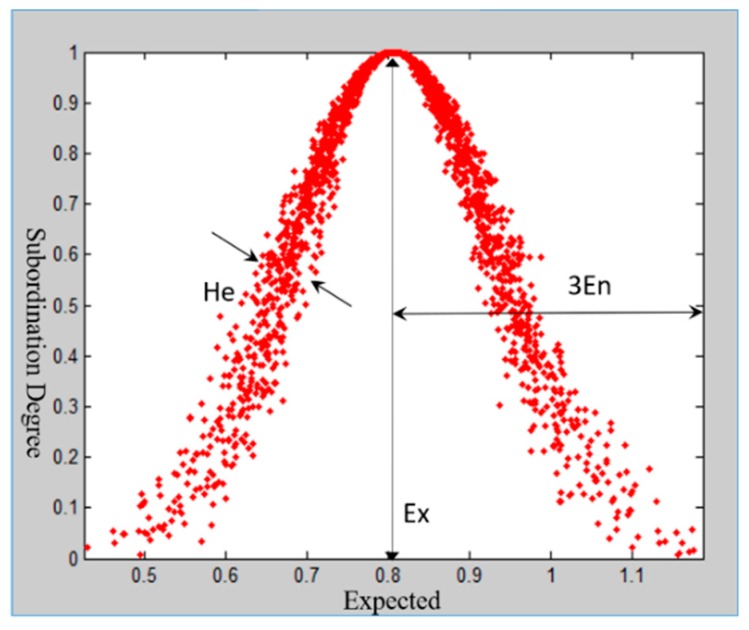
Digital characteristic map of the cloud model.

In the cloud model theory, qualitative concepts are characterized by three numerical groups: Expected $E_{x}$, Standard $E_{n}$ and Superstandard $H_{e}$. The digital characteristics of the cloud are shown in Figure 6. Here, the horizontal axis represents the scope of the uncertainty measure of the concept, and the vertical axis represents the degree of membership. Among them, we have the following: (1) $E_{x}$ is the core value of the concept, which describes qualitative concepts, namely, weight; (2) $E_{n}$ itself is a thermodynamics concept measuring the degree of confusion of physical systems; and (3) $H_{e}$ is also known as the entropy value, which is the deviation of the cloud. The amount of hyper entropy indirectly reflects the degree and thickness of the cloud’s dispersion. The thicker the cloud layer is, the lower the degree of cohesion.

Reverse cloud generator is a transformation model from quantitative values to qualitative concepts. On the basis of a certain amount of precise data $\left(x_{1},x_{2},\cdots ,x_{n}\right)$, it is transformed into a qualitative concept represented by digital features $\left(E_{x},\;E_{n},H_{e}\right)$. Its basic algorithm is as follows:

(1) Sample mean $\bar{X}=\sum_{j=1}^{n}x_{j}/n$ is calculated according to sample $x_{j}$. The absolute central moment of first-order sample is ∑j=1n|xj−X¯|n, and the sample variance is $S^{2}=\sum_{j=1}^{n}{\left(x_{j}-\bar{X}\right)}^{2}∕\left(n-1\right)$.
(2) The expected value $E_{x}=\bar{X}$_ is obtained according to step 1.
(3) The entropy $E_{n}=\sqrt{\frac{\pi }{2}}\frac{1}{n}\sum_{j=1}^{n}\left|X_{j}-E_{x}\right|$ is calculated according to step 2.
(4) The hyper entropy $H_{e}=\sqrt{S^{2}-E_{n}{}^{2}}$ is calculated according to steps 1 and 3.

## 4. Establishment of City Safety Resilience Assessment Model

### 4.1. Cloud Model Fitting

The expert scoring results are imported into the MATLAB implementation of the cloud model, and three digital features ($E_{x}$, $E_{n}$, and $H_{e}$) of the expert weighting factors are obtained using the inverse cloud model operation. The results are imported into the forward cloud model to generate the weighted cloud images. We observe the cloud agglomeration degree until the hyper entropy value is less than or equal to 0.02 [30]. At this time, the cloud agglomeration degree is high, and the weight scoring reaches a consensus. Then, the expert scoring results are output.

**Figure 7:**
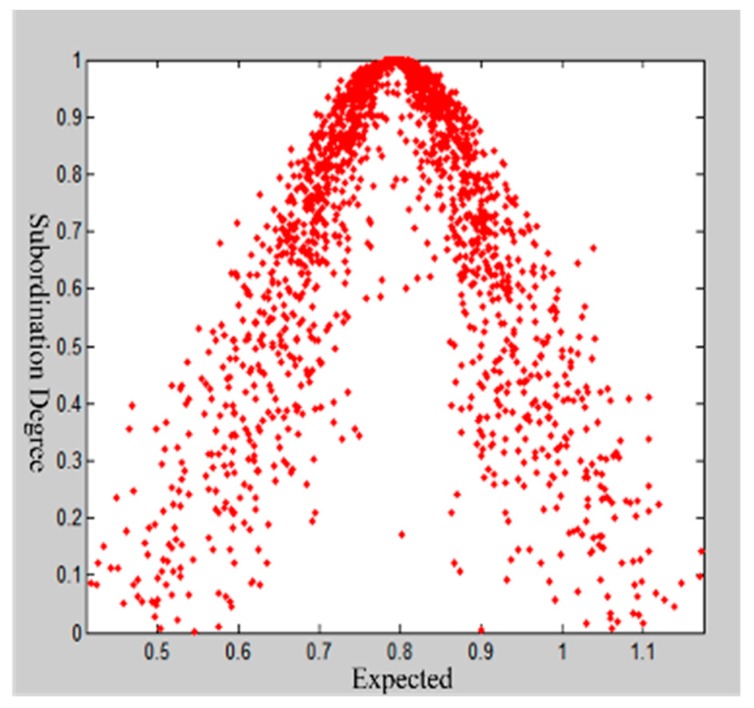
Expert score cloud model restoration chart for the first time.

**Figure 8:**
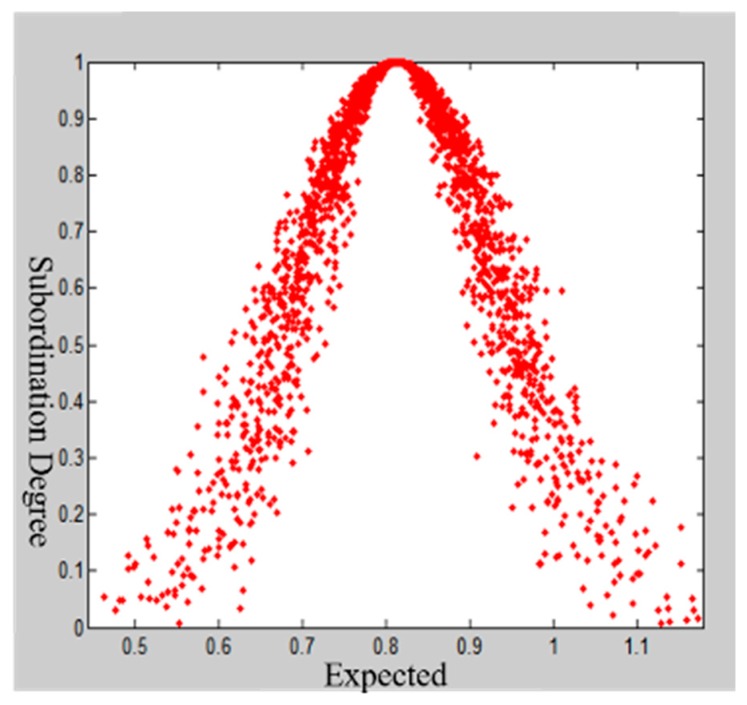
Expert scoring cloud model restoration chart for the second time.

**Figure 9:**
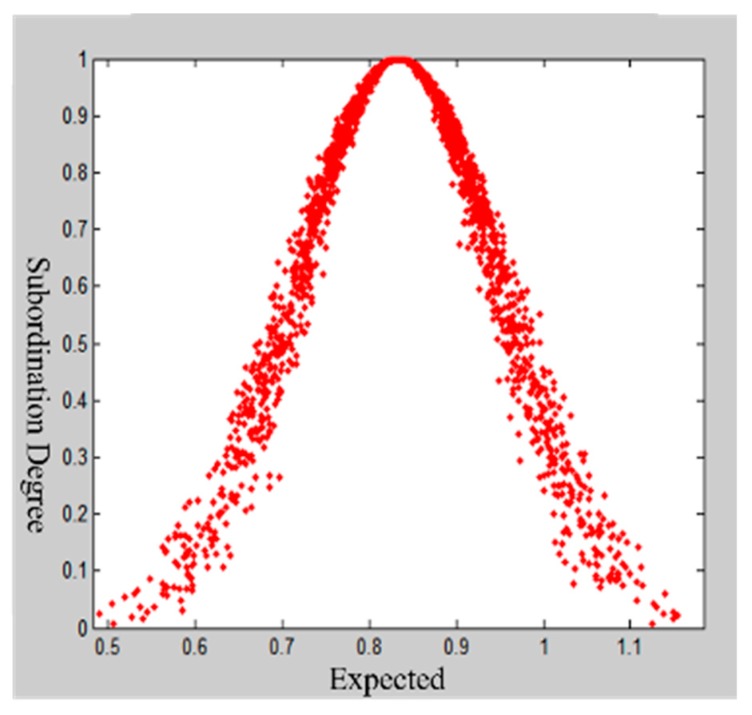
Expert scoring cloud model restoration chart for the third time.

Taking the first-level indicator “predisaster prevention” as an example, the results of the first expert scoring questionnaire are [0.7, 0.8, 1.0, 0.6, 0.9, 1.0, 1.0, 0.8, 0.8, 0.8, 0.7, 0.7, 0.8]. Using the MATLAB program, the expert scoring, the number of cloud droplets N=2000, and the three digital characteristics of cloud model are input, and the output cloud images are shown in Figure 7. Since $H_{e}=0.0410$, the cloud image is “foggy”, and the cohesion ability of the cloud image is not good. We feedback the experts’ opinions on the indicators with larger discrepancies and compare the results to the first results. The entropy values $E_{x}=0.8133$ and $E_{n}=0.1225$ and the hyper entropy $H_{e}=0.0225$ are obtained by inputting the second scored value into the MATLAB implementation. As shown in Figure 8, the cohesion of the second score nephogram is higher than that of the first one, and the entropy and super entropy are obviously reduced, but the super entropy value is still greater than 0.02. Then, we repeat the above steps. The third expert score of “Predisaster Prevention” is obtained. The cloud image that is scored by this expert assessment has high cohesion, and the hyper entropy of $H_{e}=0.0112$ is less than 0.02, as shown in Figure 9. Then, we output the weight of the index.

### 4.2. Model Fitting Results

Repeat the above process; each round will feedback the necessary information to experts and communicate, so that experts can reach a unified understanding of indicators through three questionnaires. Finally, the weight assignment of experts to 52 indicators is determined. The basic digital characteristics are shown in Table 2.

From Table 2, we can see that after three expert scorings, the $E_{n}$ and $H_{e}$ of the index’s cloud model are all small, and the cloud agglutination degree is very high. However, the $H_{e}$ values of the six indicators of “emergency support ($B_{12}$)”, “the road traffic volume and accessibility ($C_{10}$)”, “the lifeline resilience ($C_{11}$)”, “property losses Caused by Natural Disasters in Cities Every Year ($C_{22}$)”, “production accident economic losses ($C_{27}$)” and “the city emergency plan ($C_{28}$)”are still higher than 0.02, but approximately equal to 0.02. It can be considered that experts’ opinions on the expectations of indicators are basically unified and can directly output the $E_{x}$ values. Through the unification of $E_{x}$ values of each indicator, it is further demonstrated that the evaluation index system of city safety resilience designed in this study is reasonable and effective, and provides important theoretical support for the construction of resilient cities.

## 5. Result Analysis and Discussion

### 5.1. Weight Analysis and Discussion

**Figure 10:**
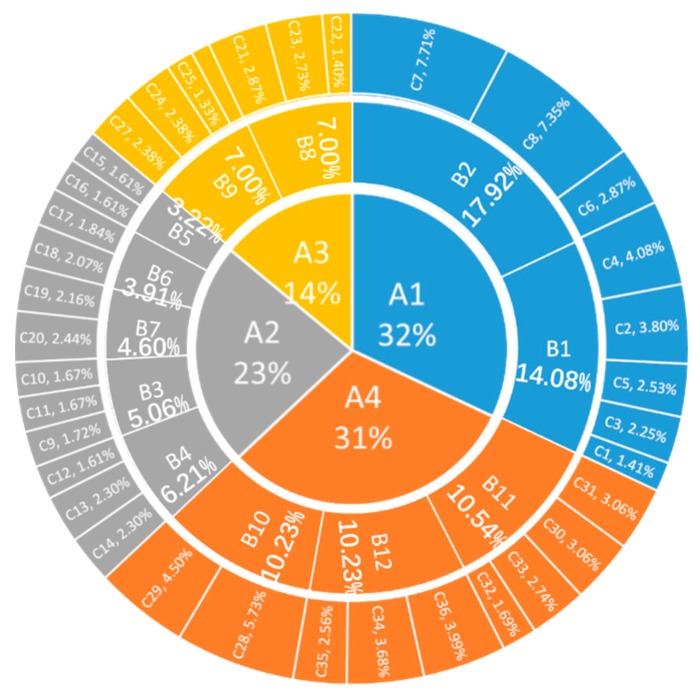
Weights of the city safety resilience index system.

The $E_{x}$ values of all indexes are obtained by fitting cloud model. The weighted average method is adopted according to the formula $W_{ij}=w_{ij}/\sum_{j=1}^{n}w_{ij}$, which can more reasonably reflect the weights of indexes. Formula: $w_{ij}$ is the j index Ex value of the i dimension, n is the number of indicators of the dimension, and the calculation results are shown in Figure 10. This weight can provide a basis for the future evaluation system of city safety resilience in China, and has a certain guiding significance for the evaluation results of city safety resilience.

(1) Among the first-level indicators, the weights of predisaster prevention (A1, 0.32) and emergency management (A4, 0.31) are close to the sum of the other two dimensions.

(2) With respect to the secondary indicators, the combination weights of grassroots city safety prevention ($B_{2}$, 0.1792), government supervision and prevention ($B_{1}$, 0.1408), the emergency management mechanism ($B_{11}$, 0.1054), the emergency plan ($B_{10}$, 0.1023) and emergency support ($B_{12}$, 0.1023) in the dimension of pre-disaster prevention account for 60% of the index system, and the results of (1) are further confirmed. In the dimension of disaster-bearing carrier, city safety facilities (B4, 0.9067) is the most important, infrastructure (B3, 0.7467) ranks second, crowd safety protection measures (B7, 0.6867) ranks third, while the population cluster statistics (B6, 0.5600) and housing buildings (B5, 0.4733) account for a smaller proportion. In the dimension of emergencies, production accidents (B9, 0.5933) and natural disasters (B8, 0.5867) accounted for the same high proportion.

(3) Among the three-level indicators, risk assessment measures (C4, 0.8467) accounted for the highest proportion, and the second highest proportion was the flood control and drainage system (C9, 0.8400).

Strengthening predisaster prevention and emergency management is more effective for improving the overall level of city safety resilience. Governments and departments at all levels should give priority to disaster prevention and mitigation and reduce the occurrence of production accidents, and promote the active participation of all sectors of the grass-roots society, creating a good social atmosphere for comprehensive work. Governments and departments at all levels should improve the emergency response mechanism, improve the system of laws, regulations and plans, increase capital investment, enhance the ability of scientific and technological support, consolidate safety and put it in the construction of facilities and equipment, strengthen the construction of material reserve network at the central-provincial-municipal-county level, do a good job in planning implementation, safe production and natural disaster assessment, and strengthen the construction of materials reserve network at all levels. Safety and tenacity culture construction. At the grass-roots level, efforts should be made to improve residents’ self-rescue ability and self-protection awareness, make use of knowledge, innovation and education, strengthen the construction of talents and professional teams, and ensure the process construction in the emergency management process.

### 5.2. Application Analysis and Discussion

Referring to Table 1 of Section 2.3, the related research on city safety resilience, and the relevant data of city safety in China in recent years, the scoring criteria involved are collected and extracted, and the scoring criteria for each evaluation index of city safety resilience are compiled. The evaluation scores of city safety resilience are obtained using the Formula (3) weighting calculation.

(3) $$R=\overset{36}{\underset{i=1}{\sum }}w_{i}\times c_{i}$$

R: City safety toughness evaluation score, which is a percentage system;
$w_{i}$: index weight;
$c_{i}$: The score result of percentage system for each index.

On the basis of referring to the construction standards of some resilient communities and resilient cities, this paper divides the value of R into five value ranges from small to large, which are, in turn: unqualified, very poor, general, good and excellent, as shown in Table 3.

If the result of city safety resilience evaluation is “unqualified”, it shows that the city has higher risk and slower recovery ability, and the city safety resilience level needs to be improved. We should strengthen the construction of city safety resilience as soon as possible, and focus on the construction of predisaster prevention and emergency management.

If the result of city safety resilience is “very poor”, it shows that the city safety resilience level is poor and the score of each index is relatively low. We should pay attention to strengthening the indicators with low construction scores.

If the result of city safety resilience evaluation is “general”, it shows that the city safety resilience level is in a normal state, and it can maintain the normal operation of city function. However, there are still some problems in preventing, responding to, or dealing with emergencies. Through the scores of each index, we can find the weak links and identify the parts that need to be improved.

If the result of city safety resilience evaluation is “better”, it shows that the safety and resilience level of the city is in the upper middle level. The construction of city safety is relatively perfect. The details of indicators still need to be strengthened.

If the result of city safety resilience evaluation is “excellent”, it shows that the city safety resilience level of the city is very high, and only needs to maintain the current level. At the same time, we should keep abreast of the changes of national policies, keep abreast of the changes of the times and constantly update and develop.

## 6. Conclusions

(1) In view of the two directions of natural disasters and production safety, the evaluation index model of city safety resilience is constructed, which includes four dimensions of predisaster prevention, disaster-bearing carrier, emergencies, emergency management, 12 secondary indicators and 36 third-level indicators. The index system of the model is relatively perfect, and the weights of each evaluation index are effectively unified.

(2) According to the weight of each evaluation index, we should take appropriate measures to improve city safety resilience. According to the weight, grading standard and Formula (2) of each evaluation index, we can get the score of city safety resilience evaluation, and help to objectively evaluate the ability of the city to recover the original city functional level quickly. The above results provide necessary data and theoretical support for Chinese city safety planners and managers.

Because of the smaller number of experts, the complexity of the index system and the smaller number of quantitative indicators, there is a certain subjectivity in the scoring of the evaluation system, which is not objective or specific enough. The next step is to improve the index system and increase the objectivity of the index system.

## Figures and Tables

**Table 1:** Reference Basis for Selecting Indicators.

Classification	Name
Categories of laws, regulations and conventions	Law of the China on Response to Emergencies [16] National Emergency Response Plan for Public Emergencies [17] Opinions of the State Council of the Central Committee of the Communist Party of China on Promoting the Reform of the System and Mechanism of Disaster Prevention, Mitigation and Relief [18] Regulations on Emergency Response to Production Safety Accidents [19] Hyogo Framework for Action 2005–2015 [20] Sendai Disaster Risk Reduction Framework 2015–2030 [21]
Indicator System Category	Indicator System Category: Manual of Strategic Performance Indicators for China’s Urban Development [22] Outline of Science and Technology for China’s Sustainable Development [18] Rockefeller Foundation Toughness City Index Research [8] UN-Habitat Urban Prosperity Index [23]
Documentation	The World Cities Report 2016 [23] Modern City Safety Space Construction Theory [15]

**Table 2:** Indicators’ digital characteristics.

First-Level Indicators	$E_{x}$	$E_{n}$	$H_{e}$	Secondary Indicators	$E_{x}$	$E_{n}$	$H_{e}$	Tertiary Indicators	$E_{x}$	$E_{n}$	$H_{e}$
Predisaster Prevention ($A_{1}$)	0.8333	0.1170	0.0112	government supervision and prevention ($B_{1}$)	0.5867	0.1682	0.0091	the city safety supervision department ($C_{1}$)	0.2800	0.1604	0.0163
								the active cooperation of different departments ($C_{2}$)	0.7933	0.1615	0.0169
								the city safety management laws and regulations ($C_{3}$)	0.4667	0.1393	0.0115
								risk assessment measures ($C_{4}$)	0.8467	0.1125	0.0024
								a risk reduction plan ($C_{5}$)	0.5467	0.1047	0.0165
				grassroots city safety prevention ($B_{2}$)	0.7467	0.1883	0.0087	emergency prevention regulations ($C_{6}$)	0.3067	0.1270	0.0159
								the propaganda of emergency prevention ($C_{7}$)	0.8200	0.1404	0.0141
								the categories and quantities of safety equipment and facilities ($C_{8}$)	0.7933	0.1615	0.0169
Disaster-bearing Carrier ($A_{2}$)	0.6097	0.1092	0.0132	Infrastructure ($B_{3}$)	0.7467	0.2061	0.0138	the flood control and drainage system ($C_{9}$)	0.8400	0.0969	0.0179
								the road traffic volume and accessibility ($C_{10}$)	0.7867	0.1225	0.0225
								the lifeline resilience ($C_{11}$)	0.7867	0.1225	0.0225
				city safety facilities ($B_{4}$)	0.9067	0.0780	0.0173	the number of hospitals ($C_{12}$)	0.5667	0.1337	0.0149
								fire protection coverage ($C_{13}$)	0.7667	0.1170	0.0112
								the number of city shelters ($C_{14}$)	0.7667	0.1393	0.0115
				housing buildings ($B_{5}$)	0.4733	0.1526	0.0151	the number of anti-seismic buildings ($C_{15}$)	0.3200	0.1471	0.0094
								the number of old residential areas ($C_{16}$)	0.3200	0.1136	0.0125
				the population cluster statistics ($B_{6}$),	0.5600	0.1838	0.0145	the population cluster locations ($C_{17}$)	0.7533	0.1047	0.0165
								the population density ($C_{18}$)	0.8333	0.1393	0.0115
				crowd safety protection measures ($B_{7}$)	0.6867	0.1384	0.0145	the crowd evacuation guidelines ($C_{19}$)	0.6867	0.1348	0.0145
								the crowd safety facilities ($C_{20}$)	0.7733	0.1615	0.0169
Emergencies ($A_{3}$)	0.3667	0.1170	0.0112	natural disasters ($B_{8}$)	0.5867	0.1181	0.0122	the frequency of natural disasters in city areas ($C_{21}$)	0.8333	0.1393	0.0165
								property losses Caused by Natural Disasters in Cities Every Year ($C_{22}$)	0.4133	0.1225	0.0225
								casualties Caused by Natural Disasters in Cities Every Year ($C_{23}$)	0.8067	0.1426	0.0182
				production accidents ($B_{9}$)	0.5933	0.1462	0.0182	the frequency of production accidents ($C_{24}$)	0.7667	0.1393	0.0115
								the mortality rate per 100,000 people as a result of production accidents ($C_{25}$),	0.4267	0.1526	0.0151
								the accident GDP per 100 million yuan based on the mortality rate ($C_{26}$)	0.3067	0.1615	0.0169
								production accident economic losses ($C_{27}$)	0.7800	0.1303	0.0209
Emergency Management ($A_{4}$)	0.8200	0.1738	0.0090	the emergency plan ($B_{10}$)	0.8400	0.1103	0.0202	the city emergency plan ($C_{28}$)	0.7867	0.1393	0.0201
								the city emergency related laws and regulations ($C_{29}$)	0.6075	0.1067	0.0027
				the emergency management mechanism ($B_{11}$)	0.8800	0.1003	0.0153	the decision-making and disposal mechanism ($C_{30}$)	0.8267	0.1025	0.0127
								the information reporting mechanism ($C_{31}$)	0.8267	0.1025	0.0127
								the emergency linkage mechanism ($C_{32}$)	0.4667	0.1170	0.0112
								the recovery and reconstruction mechanism ($C_{33}$)	0.7333	0.1281	0.0159
				emergency support ($B_{12}$)	0.8333	0.1727	0.0337	the number of city professional rescue workers ($C_{34}$)	0.7667	0.1281	0.0159
								the frequency of emergency drills ($C_{35}$)	0.5333	0.1170	0.0112
								the city rescue material reserves ($C_{36}$)	0.8200	0.1136	0.0152

**Table 3:** City safety resilience classification.

Total Score of Index System Evaluation	City Safety Resilience Level
0 ≤ R < 60	Unqualified
60 ≤ R < 70	Very poor
70 ≤ R < 80	General,
80 ≤ R < 90	Good
90 ≤ R < 100	Excellent
